# Machine learning-based prediction of composite risk of cardiovascular events in patients with stable angina pectoris combined with coronary heart disease: development and validation of a clinical prediction model for Chinese patients

**DOI:** 10.3389/fphar.2023.1334439

**Published:** 2024-01-10

**Authors:** Zihan Wang, Ziyi Sun, Linghua Yu, Zhitian Wang, Lin Li, Xiaoyan Lu

**Affiliations:** ^1^ Graduate School, Beijing University of Chinese Medicine, Beijing, China; ^2^ Science Faculty, University of Auckland, Auckland, New Zealand; ^3^ Department of Integrative Cardiology, China-Japan Friendship Hospital, Beijing, China

**Keywords:** stable angina pectoris, coronary heart disease, cardiovascular risk, prediction, risk models, machine learning

## Abstract

**Objective:** To develop a risk score model for the occurrence of composite cardiovascular events (CVE) in patients with stable angina pectoris (SA) combined with coronary heart disease (CHD) by comparing the modeling effects of various machine learning (ML) algorithms.

**Methods:** In this prospective study, 690 patients with SA combined with CHD attending the Department of Integrative Cardiology, China-Japan Friendship Hospital, from October 2020 to October 2021 were included. The data set was randomly divided into a training group and a testing group in a 7:3 ratio in the per-protocol set (PPS). Model variables were screened using the least absolute shrinkage selection operator (LASSO) regression, univariate analysis, and multifactor logistic regression. Then, nine ML algorithms are integrated to build the model and compare the model effects. Individualized risk assessment was performed using the SHapley Additive exPlanation (SHAP) and nomograms, respectively. The model discrimination was evaluated by receiver operating characteristic curve (ROC), the calibration ability of the model was evaluated by calibration plot, and the clinical applicability of the model was evaluated by decision curve analysis (DCA). This study was approved by the Clinical Research Ethics Committee of China-Japan Friendship Hospital (2020-114-K73).

**Results:** 690 patients were eligible to finish the complete follow-up in the PPS. After LASSO screening and multifactorial logistic regression analysis, physical activity level, taking antiplatelets, Traditional Chinese medicine treatment, Gensini score, Seattle Angina Questionnaire (SAQ)-exercise capacity score, and SAQ-anginal stability score were found to be predictors of the occurrence of CVE. The above predictors are modeled, and a comprehensive comparison of the modeling effectiveness of multiple ML algorithms is performed. The results show that the Light Gradient Boosting Machine (LightGBM) model is the best model, with an area under the curve (AUC) of 0.95 (95% *CI* = 0.91–1.00) for the test set, Accuracy: 0.90, Sensitivity: 0.87, and Specificity: 0.96. Interpretation of the model using SHAP highlighted the Gensini score as the most important predictor. Based on the multifactorial logistic regression modeling, a nomogram, and online calculators have been developed for clinical applications.

**Conclusion:** We developed the LightGBM optimization model and the multifactor logistic regression model, respectively. The model is interpreted using SHAP and nomogram. This provides an option for early prediction of CVE in patients with SA combined with CHD.

## 1 Introduction

Coronary heart disease (CHD) is one of the most common causes of death worldwide, and its clinical mortality has declined substantially in recent years due to the growth in the level of diagnosis and treatment (Virani et al., 2021). However, under this condition, the incidence of stable angina (SA) due to increased myocardial load is still on the rise, at about 7.7% (Abbasi et al., 2018). Patients with SA alone are currently considered to be at low risk of cardiovascular events (CVE) (Fladseth et al., 2022), but the risk of CVE is significantly higher when they have a combination of definite coronary artery disease (CAD) (Barbero et al., 2016).

The occurrence of CVE is influenced by a combination of factors, and a comprehensive assessment to optimize management strategies for patients with different needs is particularly important, while effective risk assessment to predict the course of the disease is a key initiative to prevent CVE (Akinosun et al., 2021). Constructing risk models based on complex clinical characteristics can predict the probability of future risk of important clinical outcomes in specific patient populations, and accurate risk prediction models help clinicians develop patient-specific management measures (Damluji et al., 2021). Several cardiovascular disease risk prediction models have been published in international guidelines (Woodward et al., 2007; D’Agostino et al., 2008; Goff et al., 2014), but these data are mostly derived from Western samples, and regional differences in cardiovascular disease spectrum and prevalence factors make them not very applicable to the Chinese population. Therefore, it is urgent to develop risk prediction models that meet the disease characteristics of Chinese patients. Machine learning (ML) algorithms are now widely used for early warning prediction of diseases and are more advantageous in handling clinical multidimensional data. Suri et al. (2022) compared the risk of bias in cardiovascular disease risk prediction between ML and non-ML algorithms. They showed that ML algorithms had lower bias and were superior in predicting death or the occurrence of a composite CVE as the outcome event. In addition, the modeling performance of different ML algorithms varies, so it is necessary to compare the modeling effectiveness of different ML algorithms, and poor model interpretability is also an unavoidable problem of ML (Ahsan and Siddique, 2022).

The Seattle Angina Questionnaire (SAQ) is a useful tool for reporting the health status of patients with CHD and is widely used in clinical practice, but the interpretation of the results of this questionnaire is slightly complicated and there are no clear criteria for assessing the prognosis of patients (Thomas et al., 2021). Based on this, this study intends to construct a risk score model for predicting the probability of developing composite CVE in patients with SA comorbid with CHD using clinical characteristics such as patients’ SAQ from Chinese data sources. Create the best model and the model with the best interpretation by comparing the modeling effectiveness of multiple ML algorithms. This provides a reliable and convenient method for determining the prognosis of patients with SA combined with CHD.

## 2 Materials and methods

### 2.1 Selection methods

This study is a prospective cohort study and follows Tripod reporting specifications (Collins et al., 2015). A total of 827 patients with SA combined with CHD who visited the Department of Integrative Cardiology, China-Japan Friendship Hospital from 2020-10 to 2021-10 were screened. Patients in whom data collection was affected by psychological or language factors; those who developed heart failure; those with severe systemic diseases such as tumors; or those with an expected survival time of <12 months were excluded. Finally, 104 patients were excluded and 723 patients were included in the study. The specific patient inclusion flow is shown in Supplementary Figure S1.

The study was conducted in accordance with the tenets of the Declaration of Helsinki, and all patients provided informed consent allowing the use of the data for further clinical studies. The study protocol was approved by the Clinical Research Ethics Committee of China-Japan Friendship Hospital (2020-114-K73) and registered with the China Clinical Trial Registry (ChiCTR1800017891).

### 2.2 Diagnostic criteria

The 1979 Nomenclature and Diagnostic Criteria for Ischemic Heart Disease jointly published by the International Society of Cardiology and the World Health Organization (National Institutes of Health, 1979) and the 2012 Guidelines for the Diagnosis and Treatment of Stable Ischemic Heart Disease jointly published by the American Heart Association and other societies (Fihn et al., 2012) were referred to.

### 2.3 Research methods

#### 2.3.1 Basic information

Basic information of the study population was collected, including demographic information (sex, age, height, weight); other relevant medical history (hypertensive disease, diabetes, hyperlipidemia, carotid atherosclerosis, stroke, renal insufficiency); and personal habits (smoking, alcohol consumption, and daily physical activity). Daily physical activity was judged by the patients on their own merits. Low or high-physical activity was respectively classified based on 60 min of physical activity of moderate intensity or more per day.

#### 2.3.2 Testing and examination

All patients had venous blood specimens collected on an empty stomach in the early morning of the second day after admission, and homocysteine (Hcy), low-density lipoprotein cholesterol (LDL-C), lipoprotein-a (Lp-a), glycated hemoglobin (HbA1c), serum urea nitrogen (urea), and serum creatinine (Scr) were measured at the hospital’s Department of Laboratory Medicine. All patients had undergone coronary angiography. Their latest examination results were retrieved from the medical record system of the hospital: the lesions in different vessels were recorded, and the final Gensini score was calculated according to the Gensini Score Calculation Guidelines (Rampidis et al., 2019). All patients underwent coronary angiography performed by the same team of skilled specialists.

#### 2.3.3 Medications

In this study, no medication interventions were carried out, and the treatment regimen was based on the prescription of the patient’s primary care physician. The use of antiplatelet agents (aspirin or clopidogrel); anticoagulants (warfarin, dabigatran, rivaroxaban); antianginal drugs (trimetazidine, nicorandil); vasodilators (nitrate ester); angiotensin-converting enzyme inhibitors (ACEI) or angiotensin receptor blockers (ARB); β-blockers; calcium channel blockers (CCB); lipid-lowering drugs (statins or ezetimibe); and traditional Chinese medicine (including herbal tonics and proprietary Chinese medicines) was recorded.

#### 2.3.4 Living situation assessment

The 19-item SAQ was used to assess patients’ SA condition and quality of life. Information was collected from patients by an independent investigator, and five dimensions were assessed on an article-by-article basis: degree of limitation of exertional capacity, anginal stability, anginal frequency, disease perception, and treatment satisfaction, and points were calculated according to published criteria (Thomas et al., 2021).

#### 2.3.5 Follow-up and outcome

Follow-up was performed by an independent investigator, with outpatient visits or telephone follow-up every 6 months after patients were enrolled in the study to collect information over time until the 12-month study period ended. Patients who withdrew on their own, could not be contacted, or were unable to cooperate in completing follow-up visits were considered to be dislodged.

A composite CVE was used as the primary endpoint event, including nonfatal myocardial infarction, revascularization, all-cause death, readmission for angina attack or heart failure or malignant arrhythmia, and stroke.

#### 2.3.6 Sample calculation

The initial variables were selected based on expertise and clinical experience, and certain similar or related variables were combined (e.g., fasting plasma glucose and HbA1c). In the previous study, which confirmed a high variability in association with the outcome, these variables have also been included in our study. A total of 35 variables were included in the study as factors that could potentially influence prognosis. The final study cohort included 723 patients with SA with CHD, in line with the principle that each variable corresponds to approximately ten patients in the regression analysis (van Smeden et al., 2019).

### 2.4 Statistical analysis

#### 2.4.1 Missing value handling

Patients who could eventually complete the follow-up among those included in the study were selected for per-protocol analysis (PPS). Multiple interpolation was performed using the MICE package (V3.16.0) in R for variables with <20% missing values.

#### 2.4.2 Variable selection and optimal modelling

Statistical analysis and visualization were performed in R (V3.6.3) and Python (V3.7). Normally distributed data were expressed as mean ± standard deviation, and non-normally distributed data were expressed as median and quartiles, and count data were expressed as percentages. In comparing the sample means of two groups, the independent samples t-test was used for normally distributed data, the rank sum (Wilcoxon) test was used for non-normally distributed data, and the chi-square test was used for count data. *p* < 0.05 was considered statistically significant for all analyses.

The data was randomly sampled in the ratio of 7:3 and divided into training and testing sets. The variables in the training set were initially screened using the least absolute shrinkage and selection operator (LASSO) regression, and univariate and multivariate logistic regression analyses were performed on the screened variables, respectively. The statistically different (*p* < 0.05) variables in the multivariate logistic regressions were used for further model construction. After that, Python (sklearn 0.22.1, XGBoost 1.2.1, LightGBM 3.2.1) was used to build the classification multi-model for the eXtreme Gradient Boosting (XGBoost), Logistic regression, Light Gradient Boosting Machine (LightGBM), RandomForest (RF), Adaptive boosting (AdBoost), Multilayer Perceptron (MLP), support vector Machine (SVM), K-Nearest-Neighbors (KNN), and Gaussian Naïve Bayes (GNB) respectively. The training set was utilized to construct multiple classification models, and the modeling effects of different algorithms were compared. The validation set generated through ten-fold cross-validation method was employed to validate the models. The determination of the optimal model requires a combination of several metrics. Python (sklearn 0.22.1) was used to construct receiver operating characteristic curve (ROC) curves and calculate the area under the curve (AUC) to assess the accuracy of predictive model identification. R (rmda 1.6) was used to plot a decision curve analysis (DCA) to assess the clinical applicability of the model. Calibration curves were plotted using Python (sklearn 0.22.1) to measure the calibration of the model. Once the optimal model has been determined, the construction of the optimal model begins. 70% of the data was used to train the model with ten-fold cross-validation of the model and 30% of the data was used to test the model additionally.

#### 2.4.3 Interpretation tools for the model

Since most ML models are black-box models, poor model readability limits their application in clinical settings. In contrast, the nomogram built based on regression models has outstanding advantages regarding the readability of model results. Therefore, for the optimal model, we interpreted the model using Python (SHAP 0.39.0) to plot the SHapley Additive exPlanation (SHAP) plots of importance and contribution to the model. SHAP is a unified approach that provides a global understanding of the ML model, accurately calculating the contribution and impact of each feature to the final prediction. The SHAP value shows how each predictor positively or negatively affects the target variable. Each observation in the dataset can also be interpreted in terms of a specific set of SHAP values (Lundberg and Lee, 2017). For the multifactorial logistics regression model, we interpreted the model using a nomogram and further developed an online risk calculator based on the R/Shiny webpage (https://wzhprediction.shinyapps.io/My_DynNomapp/). Finally, we determined whether the patients had CVE based on the model scores, compared them with the actual situation, and calculated the corresponding sensitivity, specificity, positive predictive value, negative predictive value, and F1 score for both models.

## 3 Results

### 3.1 Study population

We excluded 33 (4.6%) patients who did not complete follow-up. In the final PPS consisting of 690 patients with complete data available, we randomly divided the patients into a training group (*N* = 488) and a testing group (*N* = 202) in a ratio of 7:3. The baseline characteristics of the two groups of patients were similar (Supplementary Table S1). At the end of the study follow-up, the cumulative primary outcome CVE occurred in 56 patients (8.1%), with similar outcomes for patients in the training and testing groups (both *p* > 0.05).

### 3.2 Filtering variables

**FIGURE 1:**
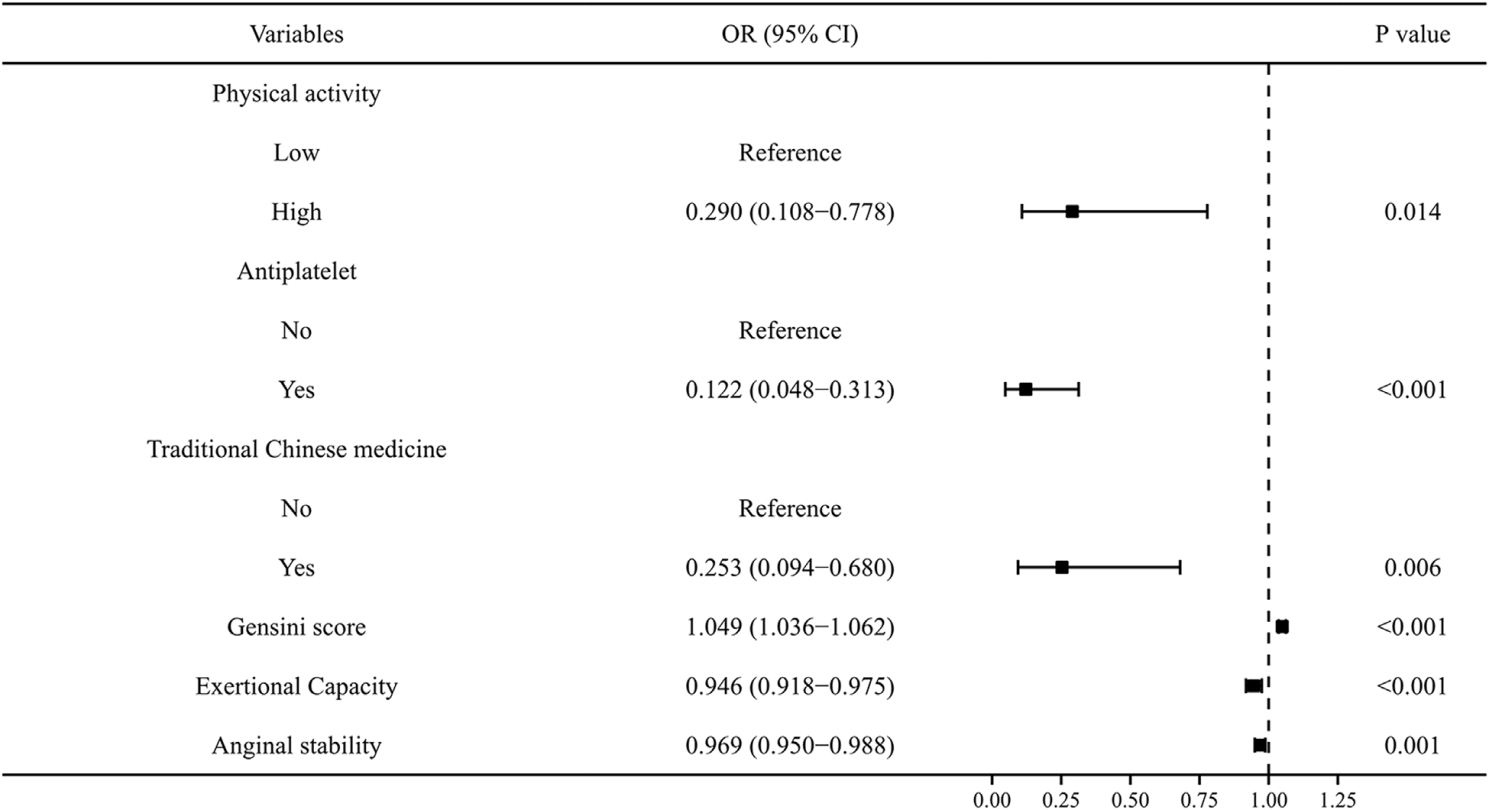
A forest diagram showing the impact of six variables on CVE.

To exclude overfitting and multicollinearity among variables, LASSO regression analysis was used to perform preliminary variables screening in the training group (Supplementary Figure S2). With the compression of coefficients, 11 variables—Physical activity, Smoking, Alcohol consumption, Renal insufficiency, Antiplatelet, Traditional Chinese medicine, Gensini score, Lp-a, Exertional Capacity, Anginal stability, and Treatment satisfaction—were finally selected for the next step of regression analysis. Univariate and multivariate logistic regressions were used for subsequent variables screening (Table 1), and the *p* values served as the ultimate criterion for variable selection in regression analysis; six variables, namely, Physical activity, Antiplatelet, Traditional Chinese medicine, Gensini score, Exertional capacity, and Anginal stability, were finally included for model development. The results are summarized in a forest plot (Figure 1), showing the independent correlation of each predictor for patient outcomes. Some intensity of activity (OR = 0.290, 95% *CI* = 0.108–0.778); taking antiplatelet agents (OR = 0.122, 95% *CI* = 0.048–0.313); and Chinese medicine therapy (OR = 0.253, 95% *CI* = 0.094–0.680) were protective factors for the development of CVE in patients (all *p* < 0.05), while higher Gensini score (OR = 1.049, 95% *CI* = 1.036–1.062) and lower Exertional Capacity score (OR = 0.946, 95% *CI* = 0.918–0.975) and Anginal stability score (OR = 0.969, 95% *CI* = 0.950–0.988) were risk factors for the development of CVE in patients (all *p* < 0.05).

**TABLE 1:** Logistic regression of univariate and multifactor.

Variable	Univariate analysis		Multivariate analysis	
	OR (95% CI)	*p*-value	OR (95% CI)	*p*-value
Physical activity				
Low	Reference			
High	0.358 (0.184–0.695)	0.002	0.290 (0.108–0.778)	0.014
Smoking				
No	Reference			
Yes	1.178 (0.651–2.131)	0.589		
Alcohol consumption				
No	Reference			
Yes	0.605 (0.337–1.087)	0.093		
Renal insufficiency				
No	Reference			
Yes	3.652 (1.411–9.454)	0.008		
Antiplatelet				
No	Reference			
Yes	0.094 (0.051–0.172)	<0.001	0.122 (0.048–0.313)	<0.001
Traditional Chinese medicine				
No	Reference			
Yes	0.514 (0.282–0.936)	0.03	0.253 (0.094–0.680)	0.006
Gensini score	1.045 (1.036–1.055)	<0.001	1.049 (1.036–1.062)	<0.001
Lp-a	1.000 (0.999–1.002)	0.869		
Exertional Capacity	0.943 (0.926–0.961)	<0.001	0.946 (0.918–0.975)	<0.001
Anginal stability	0.976 (0.964–0.988)	<0.001	0.969 (0.950–0.988)	0.001
Treatment satisfaction	0.936 (0.914–0.959)	<0.001		

Lp-a, lipoprotein-a.

### 3.3 Multi-model classification construction

**FIGURE 2:**
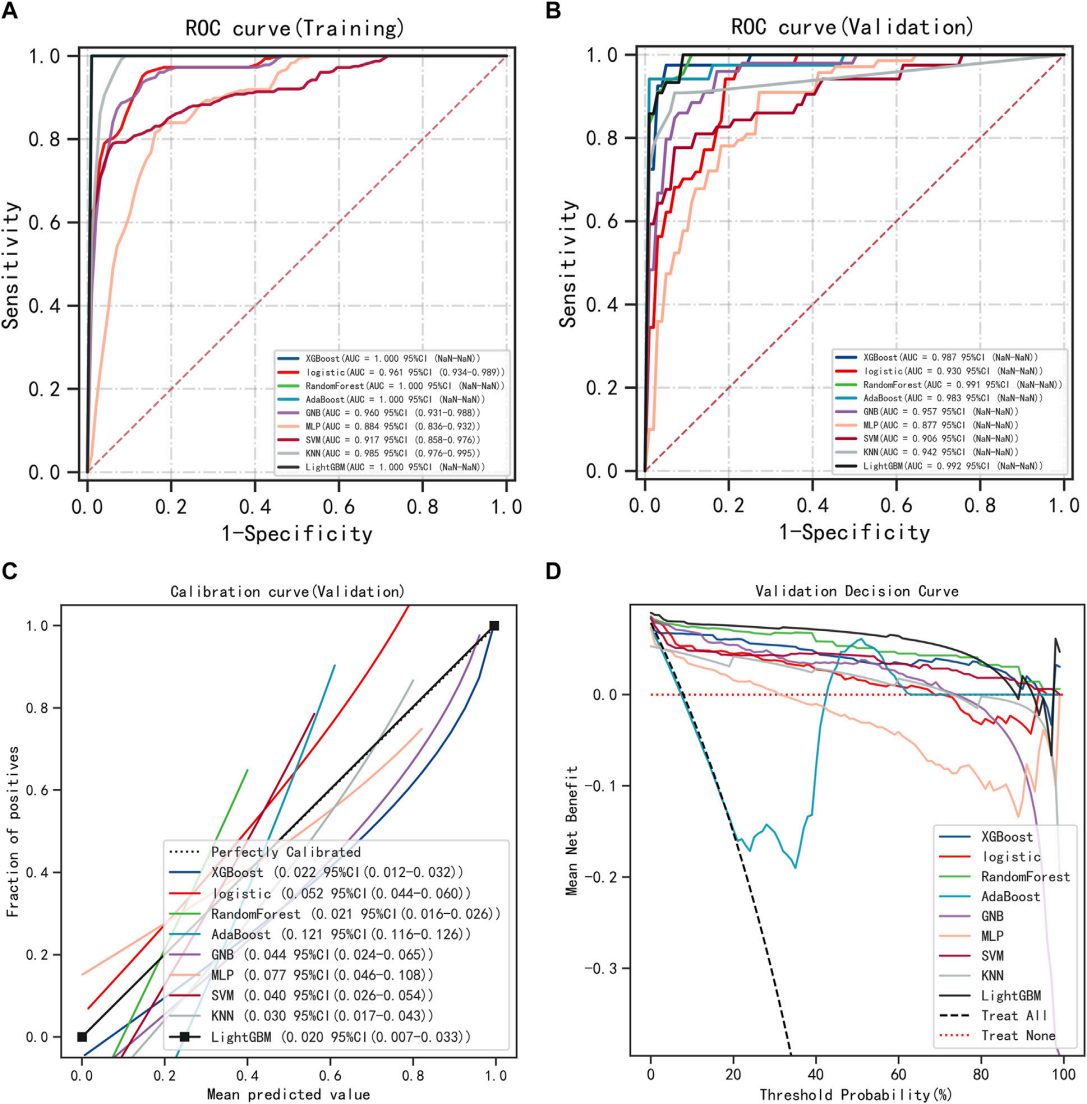
Model Accuracy for Categorical Multi-Model Evaluation. **(A)** ROC curves and AUC of the training group. **(B)** ROC curves and AUC of the validation group. **(C)** Calibration curves for the validation set. **(D)** Validation set DCA.

The six variables obtained from the multivariate logistic regression screening were used for further categorical multi-model construction, and the modeling effectiveness of the model was assessed by combining AUC, calibration, and DCA curves. The results of AUC show that the LightGBM algorithm exhibits the highest diagnostic accuracy on both the training and validation sets (Figures 2A, B). The LightGBM algorithm also showed the best accuracy for the calibration curve (Figure 2C). DCA showed good clinical applicability of LightGBM (Figure 2D). A comprehensive analysis concludes that LightGBM is the best model. The multifactor logistic regression model was also retained to plot the nomogram further. The multifactorial logistic regression model also has good diagnostic precision, accuracy, and clinical applicability but is slightly worse than LightGBM.

### 3.4 Development and validation of LightGBM and multifactor logistic regression models

**FIGURE 3:**
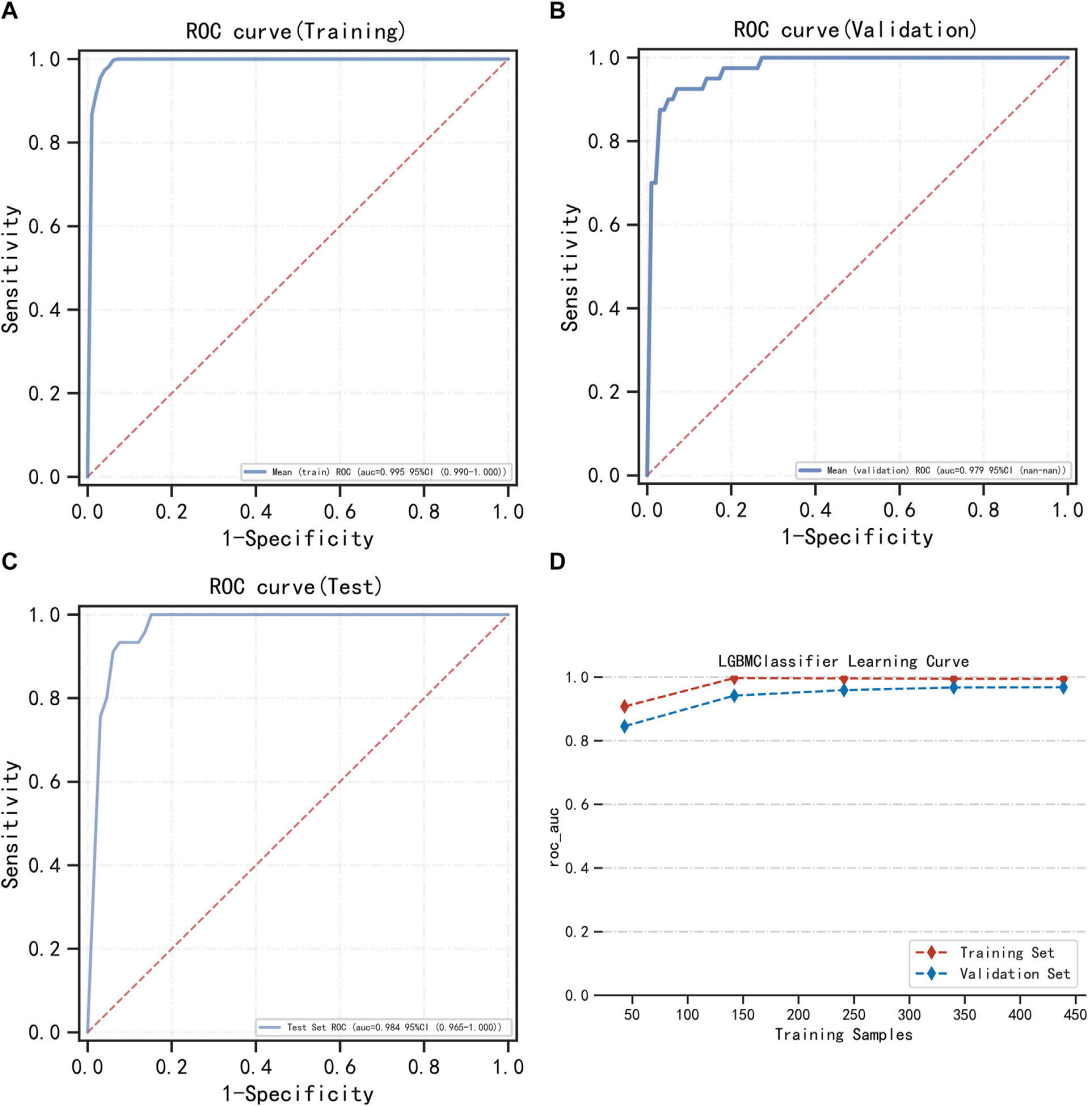
LightGBM model training, validation, and testing. **(A)** Training set of ROC curves and AUC. **(B)** Validation set of ROC curves and AUC. **(C)** Testing set of ROC curves and AUC. **(D)** Learning curve. The red dashed line represents the training set, and the blue dashed line represents the validation set.

**FIGURE 4:**
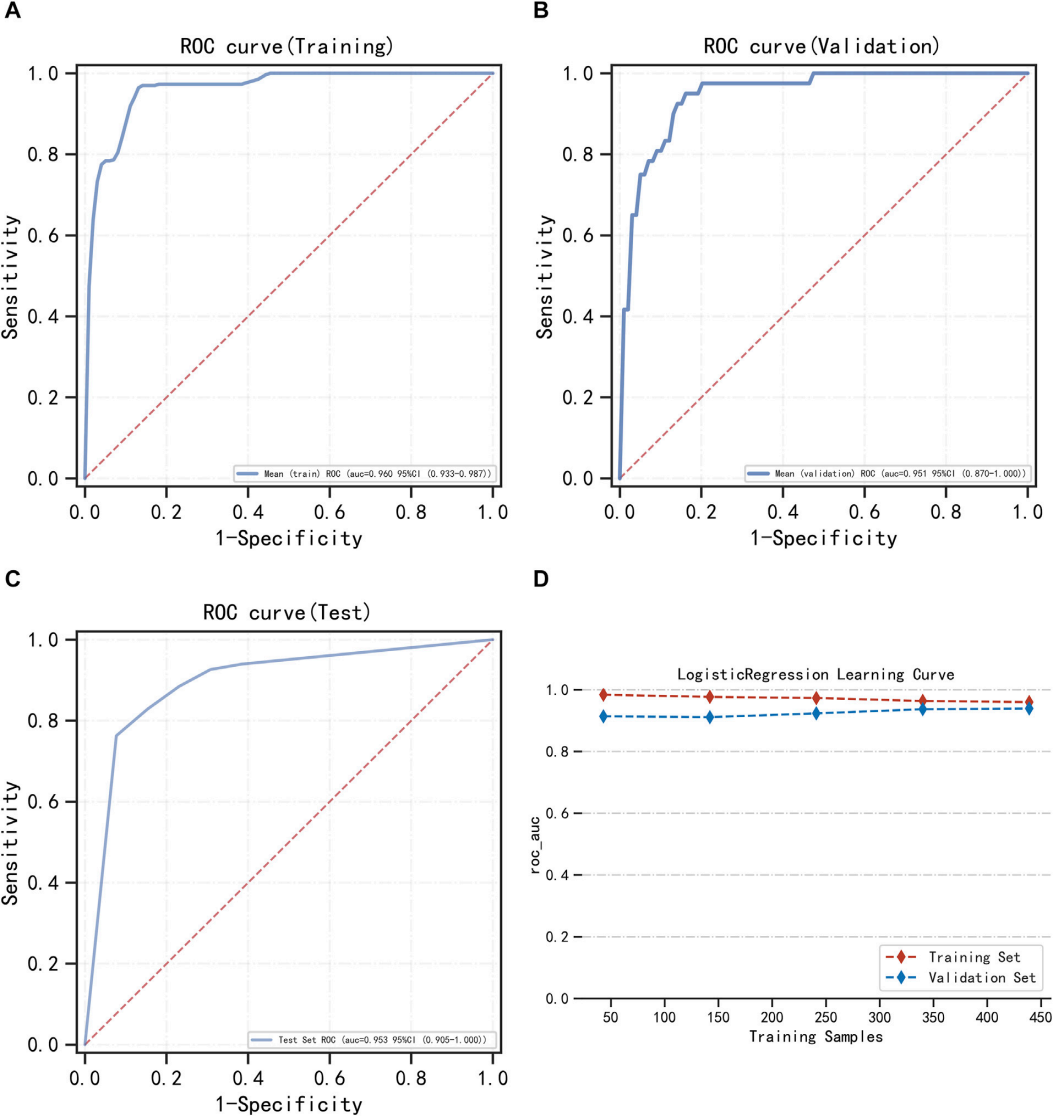
Multifactor logistic regression model training, validation, and testing. **(A)** Training set of ROC curves and AUC. **(B)** Validation set of ROC curves and AUC. **(C)** Testing set of ROC curves and AUC. **(D)** Learning curve. The red dashed line represents the training set, and the blue dashed line represents the validation set.

LightGBM and multifactor logistic regression modeling were performed in the training set, ten-fold cross-validation was performed on the data, and the performance of the models was evaluated in the testing set. The modeling results of LightGBM show an average AUC of 1.00 (95% *CI* = 0.99–1.00) for the training set, 0.98 for the validation set, and 0.98 (95% *CI* = 0.97–1.00) for the testing set (Figures 3A–C). Figure 3D shows the changes in AUC during the training process of the LightGBM model, and it can be seen that the AUC of the training set and the validation set finally stabilizes at around 1, and the model predicts well. The modeling results of the multifactor logistic regression showed an average AUC of 0.96 (95% *CI* = 0.93–0.99) for the training set, 0.95 (95% *CI* = 0.87–1.00) for the validation set, and 0.95 (95% *CI* = 0.90–1.00) for the test set (Figures 4A–C). Figure 4D shows the changes in AUC during the training process of the multifactor logistic regression model. It can be seen that the AUC of the training set and the validation set finally stabilizes at about 0.95, and the model predicts well. The consistency of the two models was evaluated using calibration plots (Supplementary Figure S3), which showed good consistency between the two models, with LightGBM being better. Finally, the clinical applicability of the models was assessed by DCA (Supplementary Figure S3), and the results again showed that both models have good clinical applicability.

### 3.5 Interpretation of models

**FIGURE 5:**
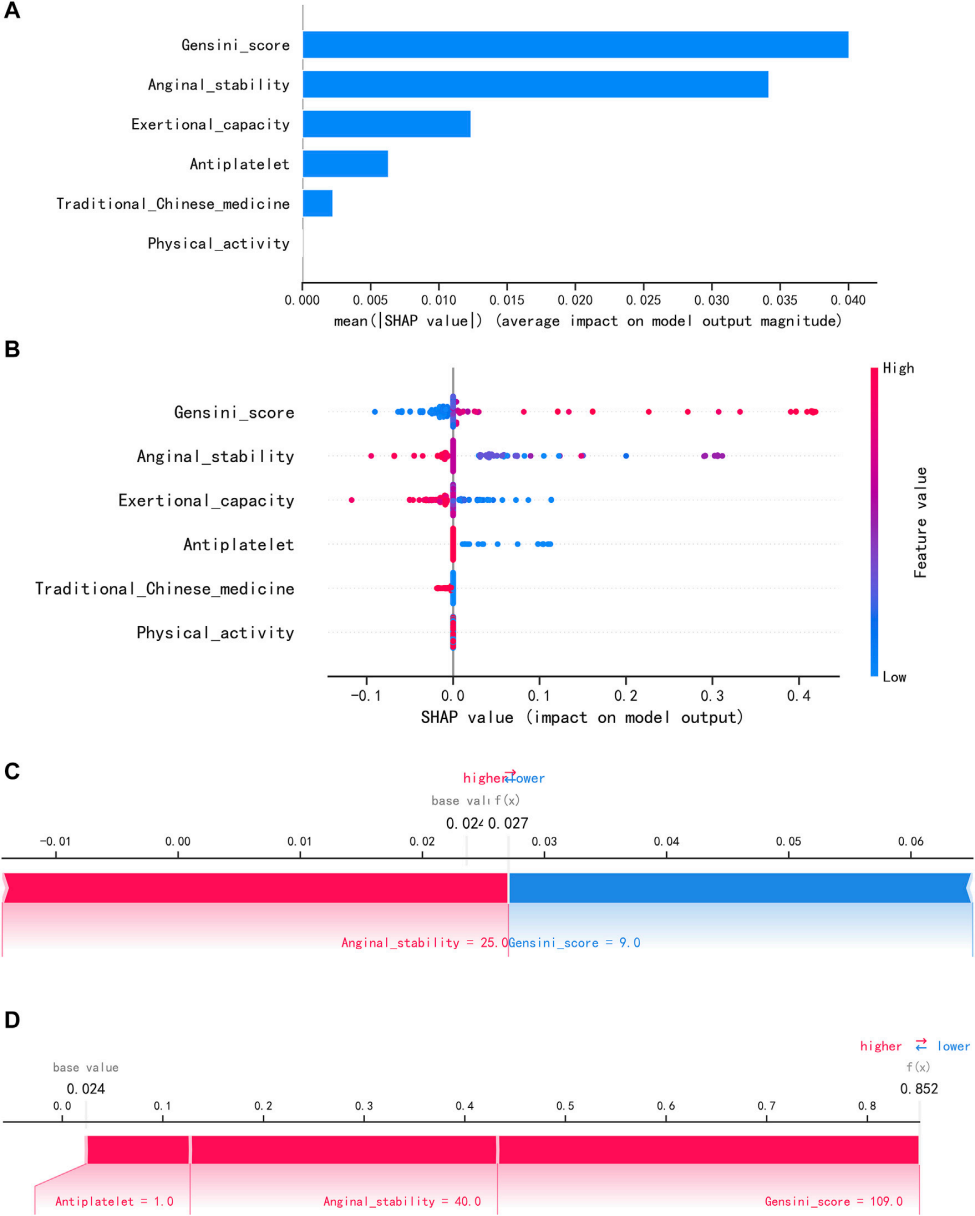
Interpretation of the LightGBM model using SHAP. **(A)** Importance ranking of features displayed by SHAP. **(B)** Characterization attributes in SHAP. **(C)** A patient who did not develop composite CVE and **(D)** a patient who develop composite CVE.

**FIGURE 6:**
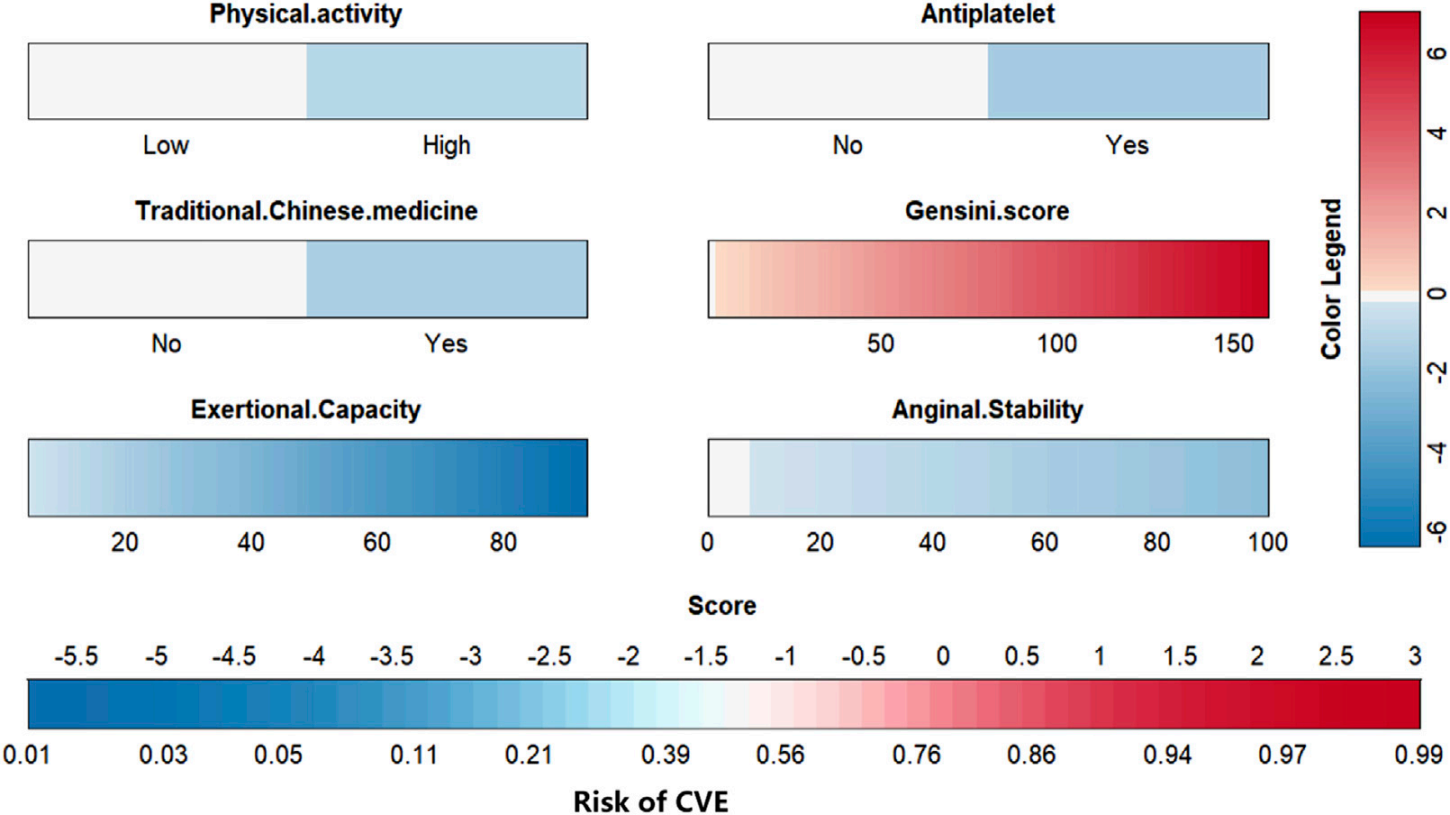
The risk scoring model was presented in a colorful nomogram.

To visually explain the LightGBM models, we use SHAP to show how the variables in these models contribute to the composite CVE. Figure 5A shows the ordering of the importance of the six variables assessed using the mean absolute value of SHAP, with the SHAP value on the x-axis indicating the importance of each covariate in the constructed model. This shows that the Gensini score is the most important predictor of future composite CVE in patients. In the summary plot of SHAP values in Figure 5B, each feature is represented along the y-axis, and the corresponding SHAP values are represented along the x-axis. These SHAP values indicate how much each feature affects the average predicted value of the model, with blue to red representing feature values from low to high. It can be seen that the higher the Gensini score, the higher the probability of a composite CVE. We also visualized individual predictions; Figure 5C shows a patient who did not develop composite CVE, and Figure 5D shows a patient who developed composite CVE. Bold numbers are probabilistic predictions [f(x)], and baseline values are predictions not entered into the model. f(x) is the log ratio for each observation. Red features indicate a facilitating effect on the occurrence of composite CVEs. In contrast, blue features are inhibitory, with longer arrows having a more significant effect on the occurrence of the outcome. For the multifactor logistic regression model, we constructed a risk score prediction model represented by a colored nomogram (Figure 6).

### 3.6 Application of predictive models

**FIGURE 7:**
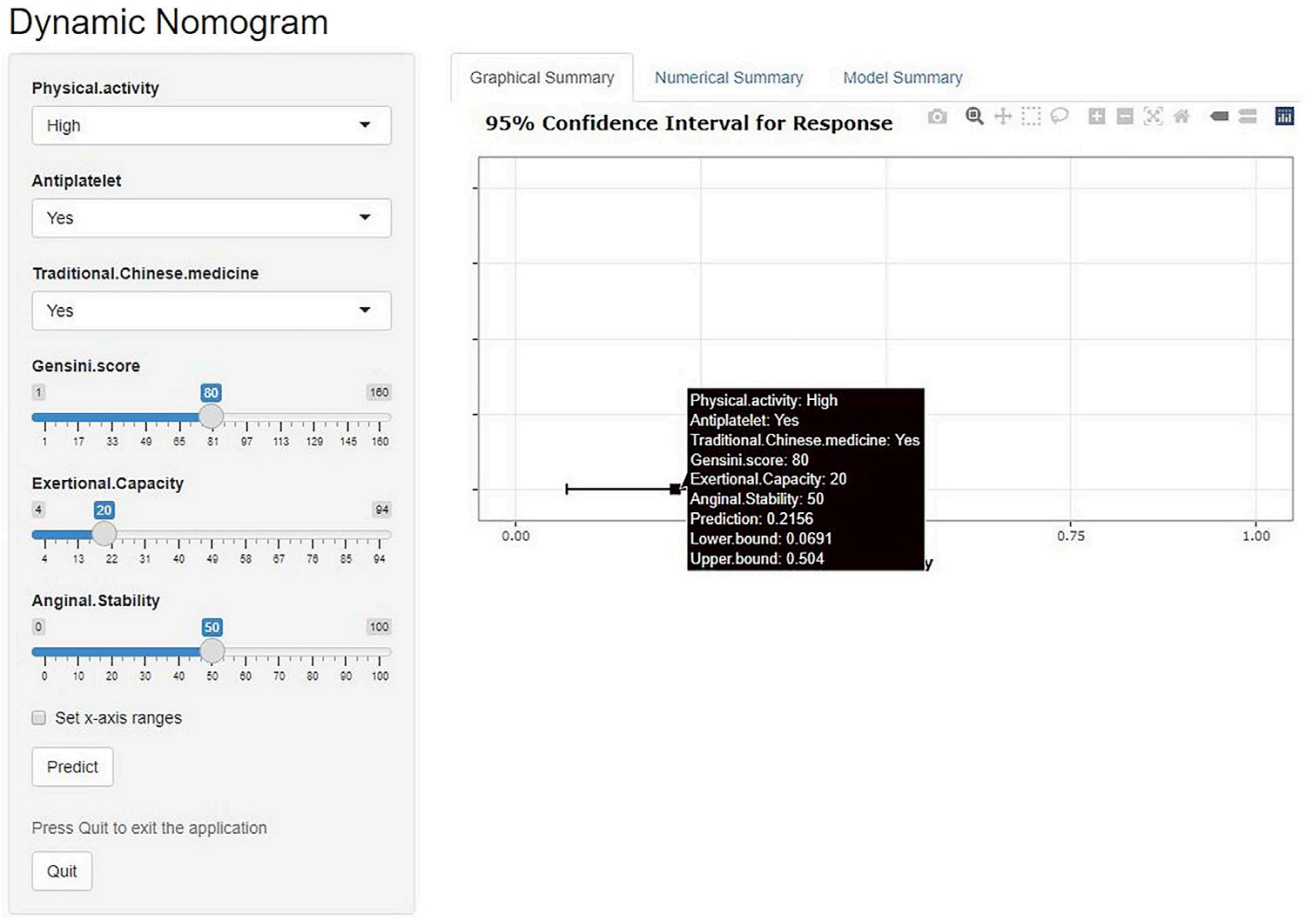
Online risk calculator based on Shiny web development. This merger aims to develop a cardiovascular events (CVE) risk prediction model for stable angina pectoris (SA) patients with coronary heart disease (CHD). Physical activity: Low or high physical activity is classified based on at least 60 min of moderate intensity exercise per day. Antiplatelet: Aspirin or clopidogrel are used as antiplatelet therapy. Traditional Chinese medicine: including herbal tonics and proprietary Chinese medicines. Gensini score: The latest results of coronary angiography are used to calculate the Gensini score. Exertional capacity: The latest results of the Seattle Angina Questionnaire (SAQ) determine the patient’s exertional capacity. Anginal stability: The latest results of the SAQ assess the patient’s anginal stability.

We have developed an online risk calculator based on multifactor logistic regression modeling on the R/Shiny webpage (https://wzhprediction.shinyapps.io/My_DynNomapp/). When this model is applied to clinical practice, the risk of CVE at 12 months of treatment in patients with SA combined with CHD can be predicted by entering the identified six variables. For example, the initial evaluation of a patient with SA combined with CHD revealed that he had good activity (Physical activity = High), was taking antiplatelets (Antiplatelet = Yes) and traditional Chinese medicine (Traditional Chinese medicine = Yes). A recent coronary angiogram showed a calculated Gensini score of 80 (Gensini score = 80) and the SAQ was calculated to obtain Exertional Capacity = 20 and Anginal stability = 50. The patient had a 21.56% probability of CVE within 12 months of treatment (Figure 7).

In addition to the network calculator described above, cut-off values, sensitivity, specificity, positive predictive value, negative predictive value, and F1 scores were calculated for both models (Table 2). The positive predictive value of LightGBM is higher than the multifactor logistics regression model. This suggests that LightGBM can help identify more positive patients, but when using the model for exclusionary diagnosis, both methods meet clinical requirements.

**TABLE 2:** Predictive performance of XGBoost and multifactor logistic regression models in different datasets.

Model	Groups	AUC	Cutoff	Accuracy	Sensitivity	Specificity	PPV	NPV	F1
LightGBM	Training set	1.00	0.14	0.96	0.99	0.96	0.66	1.00	0.79
	Validation set	0.98	0.14	0.94	1.00	0.94	0.58	0.99	0.72
	Testing set	0.98	0.13	0.96	0.93	0.95	0.71	0.98	0.80
Logistic regression	Training set	0.96	0.07	0.88	0.97	0.88	0.39	0.99	0.55
	Validation set	0.95	0.07	0.87	0.98	0.90	0.36	0.99	0.51
	Testing set	0.95	0.06	0.9	0.87	0.96	0.41	0.99	0.55

AUC, The area under the curve; PPV, positive predictive value; NPV, negative predictive value; F1: F1 score.

## 4 Discussion

Effectively preventing the occurrence of cardiovascular disease endpoint events and improving long-term treatment outcomes are the main tasks in the current prevention and treatment of CHD. Studies have shown that the combination of persistent angina in patients with CHD is strongly associated with a higher incidence of cardiovascular death or myocardial infarction (Mesnier et al., 2021). However, combining revascularization therapies with conventional medications does not provide additional clinical benefits for these patients (Stergiopoulos et al., 2014). This increasingly underscores the importance of early identification of patients with SA comorbid CHD who have a poor prognosis and echoes the call for individualized medicine. International scholars have made relevant thoughts and studies on the prevention of cardiovascular composite endpoint events in patients by establishing clinical prediction models through correlation regression analysis such as the QRISK cardiovascular disease risk algorithm (Hippisley-Cox et al., 2007), the US PCEs cohort equation (Wilson et al., 2012), the SMART risk score (Dorresteijn et al., 2013), and a 10-year risk prediction model for the development of atherosclerotic cardiovascular disease in a Chinese population (Yang et al., 2016). However, current clinical prediction models focus more on the general population without cardiovascular disease, and the relevant prognostic determinants for patients with pre-existing coronary artery disease conditions have not been adequately studied. In addition, the complexity of predictors and the high number of infrequent indicators in some studies increase the difficulty of using clinical models. ML is now increasingly being used in the early diagnosis and prediction of CHD to drive clinical decision-making. Kim et al. (2022) compared eight ML algorithms and finally developed an optimal CatBoost model for predicting obstructive CAD. The model also outperformed the established Diamond-Forest score. Few models are available for predicting adverse cardiovascular events in SA combined with CHD in the Chinese population. Therefore, based on these studies and ML techniques, we aimed to develop a new risk prediction model for patients with SA comorbid CHD based on the SAQ and general clinical information. The aforementioned issue has historically posed a significant clinical challenge that has not received due recognition.

A total of 35 clinical variables that may affect the occurrence of CVE were collected in this trial, and patients who completed the trial were included in the PPS analysis. Accordingly, 690 patients were divided into a “training group” and a “testing group” in a ratio of 7:3 based on the Tripod guidelines (Collins et al., 2015). Preliminary analysis showed that the baseline features of the training and testing groups are similar, indicating that the data obtained from random grouping possesses better homogeneity and provides higher confidence for subsequent validation. To exclude the problems of overfitting and multicollinearity among variables, we conducted the first variables screening by LASSO regression method, from which 11 independent variables related to clinical outcome events were selected, and six independent influencing factors—Physical activity, Antiplatelet, Traditional Chinese medicine, Gensini score, Exertional capacity, and Anginal stability, each of which directly affects the patient’s prognostic survival. When the model was further constructed using these six independent influences, the LightGBM model outperformed other ML models. Interpretation of the LightGBM model using SHAP highlighted that the Gensini score was the most important predictor. The model was constructed using a total of six variables, including both continuous and categorical variables. It provides a binary prediction for CVE in patients with SA combined with CHD. The diagnostic threshold is reflected in the nomogram and network calculator.

The Gensini score, calculated from the imaging results of coronary interventions, is the gold standard for diagnosing the presence of CHD in patients (Rampidis et al., 2019), and a high Gensini score is an independent risk factor for cardiac death in patients with CHD, providing valuable information for cardiovascular risk prognosis (Huang et al., 2010). Improving symptoms and quality of life is equally important in treating patients with SA. The SAQ is often used to assess the health status of SA patients (Spertus et al., 1995), where the degree of exertional capacity and anginal stability together reflect the patient’s ability to perform activities of daily living, with lower scores representing a severe decline in quality of life and strongly associated with the risk of death (Kanwar et al., 2021). Recent studies have demonstrated the importance of patient-reported signs and symptoms in predicting adverse disease outcomes, suggesting that clinicians should pay more attention to patients’ symptoms and quantitatively assess the extent of symptoms (Tripoliti et al., 2016; Parikh et al., 2023). Previous studies have shown that a certain intensity of physical activity can improve the reserve capacity of coronary blood flow and use excess body fat, effectively avoiding intravascular embolism formation and promoting the recovery of cardiac function (Pandey et al., 2018). Antiplatelet agents are recognized as an important measure that must be performed for secondary prevention in patients with CHD (Pilgrim and Windecker, 2014; Cimmino et al., 2020). Chinese medicine has been shown to reduce the incidence of CVE in patients with SA combined with CHD (Chen et al., 2021; Ge et al., 2021), and has been shown to have an ablative effect on vascular microthrombosis in several basic science studies (Jiang et al., 2020). When combined, the two drug therapies can better protect coronary vascular endothelial cells and improve microcirculatory blood flow in the heart.

The black-box nature of ML is a barrier to clinicians applying predictive modeling. Although our study utilized SHAP to quantify the significance of the variables in the model to some extent, the complexity of model interpretation will still limit its clinical application. The results provided by SHAP offer intriguing insights, highlighting the crucial role of SAQ-related assessment in patients with CHD and SA, a clinical significance that has been previously overlooked. SAQ assessment effectively captures the overall condition of patients through a range of clinical symptoms and daily life situations, offering a comprehensive quantitative evaluation method that yields abundant information. Previous studies have acknowledged the ability of SAQ to quantify functional decline and reduced quality of life caused by angina pectoris, thereby guiding future treatment strategies to some extent. It is frequently employed as an outcome evaluation tool for assessing treatment effectiveness (Ford et al., 2020). Notably, researchers have discovered a close association between Anginal stability score in the SAQ and post-coronary artery bypass grafting angina occurrence (Hattler et al., 2019), as well as its correlation with complete revascularization achievement in patients with chronic coronary artery disease (Mavromatis et al., 2023). These robust findings provide substantial support for our own results. In this study, we successfully established a link between SAQ assessment and accurate prediction of CVE risk in patients with CHD and SA—a feat not previously accomplished—emphasizing the importance for clinicians to enhance their comprehensive evaluation approach when diagnosing and treating such individuals. Multifactorial logistic regression models were also found to have better model performance in this study, and the nomogram and online calculators developed on this basis were more suitable for clinical applications, provides the reader with applied to clinical diagnosis and treatment. We retained the multifactor logistic regression model. Note that similar to the LightGBM model, the multifactor logistic regression model also has a high negative predictive value but a low positive predictive value. This suggests the need to incorporate other more specific clinical information when applying our multifactorial logistic regression model if the prediction is positive. However, when we wish to reduce the rate of underdiagnosis in high-risk populations, the model meets the demand for use. For clinicians, we offer two models depending on the user’s focus.

In conclusion, this study compared the modeling effects of different algorithms based on ML techniques. Based on full consideration of the interpretability and clinical utility of the models, LightGBM and multifactorial logistic regression models were developed for predicting the risk of CVE in patients with SA combined with CHD, respectively. The model covers the patient’s general condition, medication taking, vascular condition, and SAQ. The two models showed good sensitivity and specificity in both the training and testing cohorts, with high accuracy and clinical applicability, and played a strong role in predicting the occurrence of CVE in patients. Compared to the previous model, the present model introduces additional daily activity capacity, Gensini score, and SAQ as the main predictor variables to assess the angina attack condition and also emphasizes the impact of quality of life on patients. Moreover, the information on the incorporated variables is more accessible to collect in clinical work, the model presentation format is more intuitive and concise, and the calculation is convenient, which is conducive to generalized application.

This study has some limitations. Although the target population was Chinese patients with SA and CHD, the case source was regional, and the entire study population comprised patients from the China-Japanese Friendship Hospital, Beijing, which may have biased the collection of clinical data. While the sample size of 690 patients is not small, the cohort size can be further expanded in future studies. Second, due to the condition, we achieved external validation of the model in the testing group obtained after random partitioning, which can better demonstrate the model prediction ability if it can be validated with data from other research centers. It is also important to note that although the study mentioned the concept of “Traditional Chinese medicine,” we did not limit the specific type of medicine because of the unique and individualized treatment aspect of Chinese medicine, which may have had some influence on the results. Furthermore, we will incorporate some subsequent clinical studies and consciously optimize the existing model. For instance, clinicians may find a “low-moderate-high” risk assessment form more comfortable to use, and the clearly defined numerical criteria may also be proposed. To enhance the predictive value of the model for CVE, a larger prospective multicenter study is required with additional variables introduced, specific medication types restricted, and longer follow-up conducted. The promotion of becoming a new tool for risk assessment will be our unwavering commitment, as we diligently incorporate it into regional guidelines.

## 5 Conclusion

We conducted a prospective study on the development and validation of a clinical prediction model by including patients with SA and CHD as the study population. The clinical risk score model for predicting the occurrence of CVE was constructed based on the following variables: Physical activity, Antiplatelet, Traditional Chinese medicine, Gensini score, Exertional capacity, and Anginal stability and showed high accuracy and clinical applicability, and can be used to predict the risk of CVE in patients with SA combined with CHD. This study builds on previous literature to further the understanding of CVE risk prevention and control and provides clinicians with some evidence for diagnosis and treatment.

## Data Availability

The raw data supporting the conclusion of this article will be made available by the authors, without undue reservation.
