# Supplementary material for: Machine learning-based prediction of composite risk of cardiovascular events in patients with stable angina pectoris combined with coronary heart disease: development and validation of a clinical prediction model for Chinese patients
Source: Front Pharmacol. 2024 Jan 10;14:1334439. doi: 10.3389/fphar.2023.1334439 (PMC10806135; doi:10.3389/fphar.2023.1334439)
Supplement: Supplementary file 1 [file Table1.DOCX]

**Table S1 . Clinical characterization of the study population**

| Variable | Total (N=690) | Training group (N=488) | Testing group (N=202) | P value**^*^** |
| --- | --- | --- | --- | --- |
| Sex [male, N(%)] | 438 (63.5) | 315 (64.5) | 123 (60.9) | 0.385 |
| Age (years) | 65.1±11.32 | 65.06±10.95 | 65.20±12.20 | 0.880 |
| BMI (kg*m^-2^) | 25.57±1.69 | 25.54±1.73 | 25.64±1.60 | 0.478 |
| Physical activity [High, N(%)] | 297 (43.0) | 216 (44.3) | 81 (40.1) | 0.353 |
| Smoking [N(%)] | 427 (61.9) | 296 (60.7) | 131 (64.9) | 0.343 |
| Alcohol consumption [N(%)] | 330 (47.8) | 239 (49.0) | 91 (45.0) | 0.358 |
| Medical history [N(%)] |  |  |  |  |
| Hypertension | 280 (40.6) | 189 (38.7) | 91 (45.0) | 0.126 |
| Diabetes | 234 (33.9) | 168 (34.4) | 66 (32.7) | 0.724 |
| Hyperlipemia | 222 (32.2) | 155 (31.8) | 67 (33.2) | 0.721 |
| Carotid atherosclerosis | 245 (35.5) | 178 (36.5) | 67 (33.2) | 0.432 |
| Stroke | 78 (11.3) | 56 (11.5) | 22 (10.9) | 0.895 |
| Renal insufficiency | 28 (4.1) | 20 (4.1) | 8 (4.0) | 1.000 |
| Drugs used [N(%)] |  |  |  |  |
| Antiplatelets | 599 (86.8) | 418 (85.7) | 181 (89.6) | 0.176 |
| Antianginals | 101 (14.6) | 73 (15.0) | 28 (13.9) | 0.813 |
| Nitrate ester | 142 (20.6) | 101 (20.7) | 41 (20.3) | 1.000 |
| ACEI/ARB | 303 (43.9) | 211 (43.2) | 92 (45.5) | 0.613 |
| β-blockers | 367 (53.2) | 260 (53.3) | 107 (53.0) | 1.000 |
| CCB | 206 (29.9) | 151 (30.9) | 55 (27.2) | 0.361 |
| Anticoagulant | 62 (9.0) | 43 (8.8) | 19 (9.4) | 0.772 |
| Lipid-lowering | 611 (88.6) | 428 (87.7) | 183 (90.6) | 0.297 |
| Traditional Chinese medicine | 327 (47.4) | 229 (46.9) | 98 (48.5) | 0.738 |
| Examination |  |  |  |  |
| Gensini score | 23.00 (10.00, 47.25) | 23.00 (10.00, 48.00) | 22.00 (11.00, 45.25) | 0.648 |
| Hcy (μmol*L^-1^) | 15.00±7.39 | 15.20±8.16 | 14.51±5.07 | 0.269 |
| LDL-C (mmol*L^-1^) | 2.35±0.71 | 2.38±0.72 | 2.27±0.68 | 0.073 |
| Lp-a (mg*L^-1^) | 124.02 (56.12, 223.89) | 124.34 (56.96, 226.57) | 123.21 (47.68, 196.81) | 0.476 |
| HbA1c (%) | 6.34±2.93 | 6.40±3.39 | 6.17±1.26 | 0.349 |
| Urea(mmol*L^-1^) | 17.36±8.10 | 17.35±7.92 | 17.38±8.53 | 0.967 |
| Scr (mg*dL^-1^) | 0.96 (0.82, 1.15) | 0.96 (0.82, 1.15) | 0.96 (0.81, 1.14) | 0.664 |
| SAQ |  |  |  |  |
| Exertional capacity | 61.67±15.37 | 61.37±15.38 | 62.40±15.37 | 0.424 |
| Anginal stability | 50.67±22.13 | 51.23±22.04 | 49.33±22.34 | 0.305 |
| Anginal frequency | 75.86±20.76 | 75.33±22.00 | 77.15±17.37 | 0.294 |
| Disease perception | 65.48±17.13 | 65.36±16.99 | 65.77±17.52 | 0.774 |
| Treatment satisfaction | 80.49±11.37 | 80.77±11.40 | 79.86±11.30 | 0.338 |
| CVE [N(%)] | 56 (8.1) | 37 (7.6) | 19 (9.4) | 0.445 |

*Comparison of the difference between the training and testing groups.
