# Supplementary figures and images for: Machine learning-based prediction of composite risk of cardiovascular events in patients with stable angina pectoris combined with coronary heart disease: development and validation of a clinical prediction model for Chinese patients
Source: Front Pharmacol. 2024 Jan 10;14:1334439. doi: 10.3389/fphar.2023.1334439 (PMC10806135; doi:10.3389/fphar.2023.1334439)

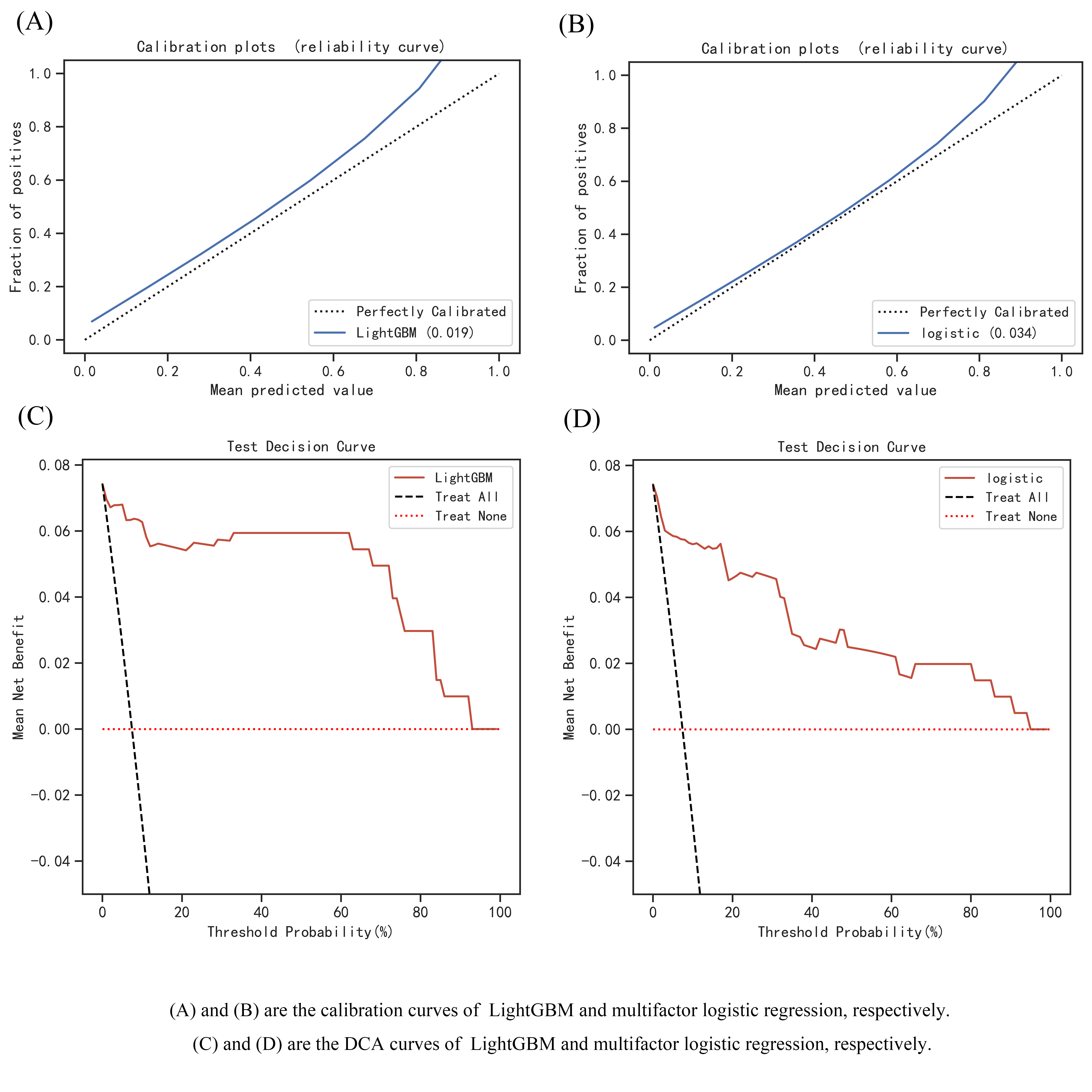

Supplement: Supplementary file 2 [file Image3.TIF]

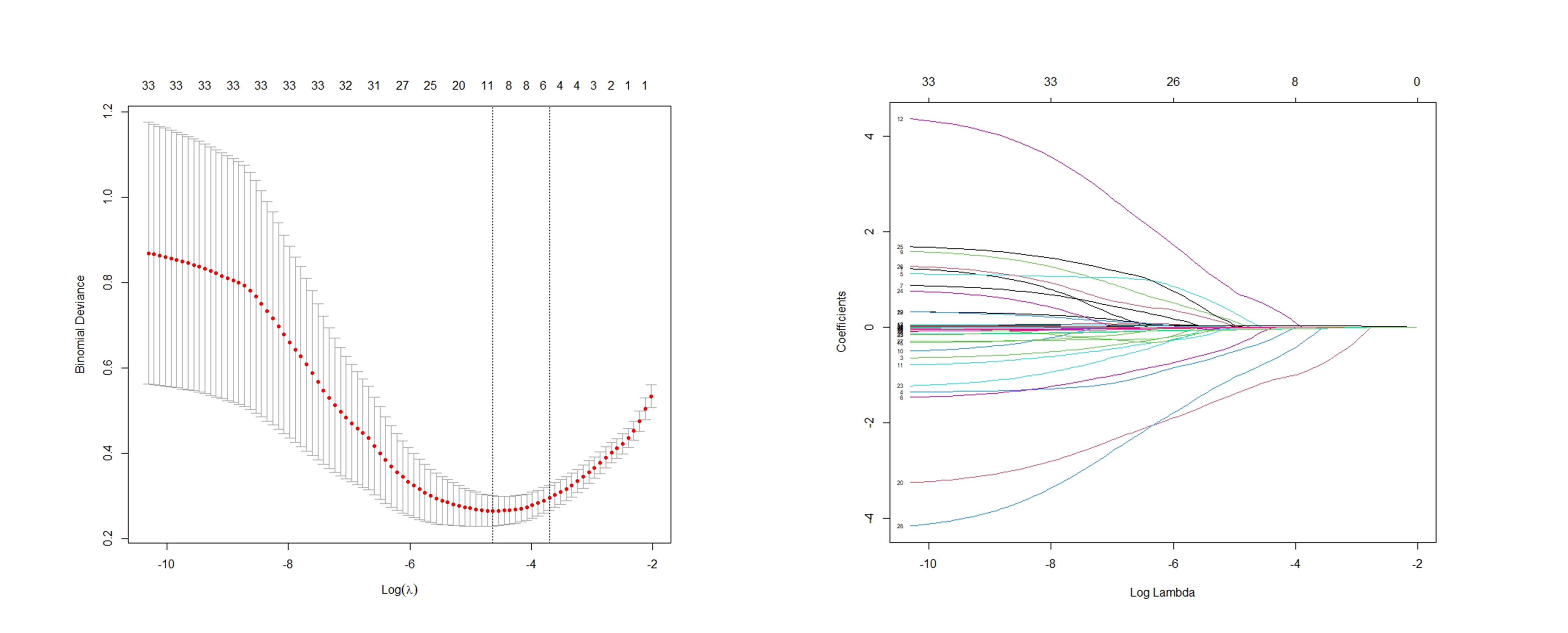

Supplement: Supplementary file 4 [file Image2.TIF]

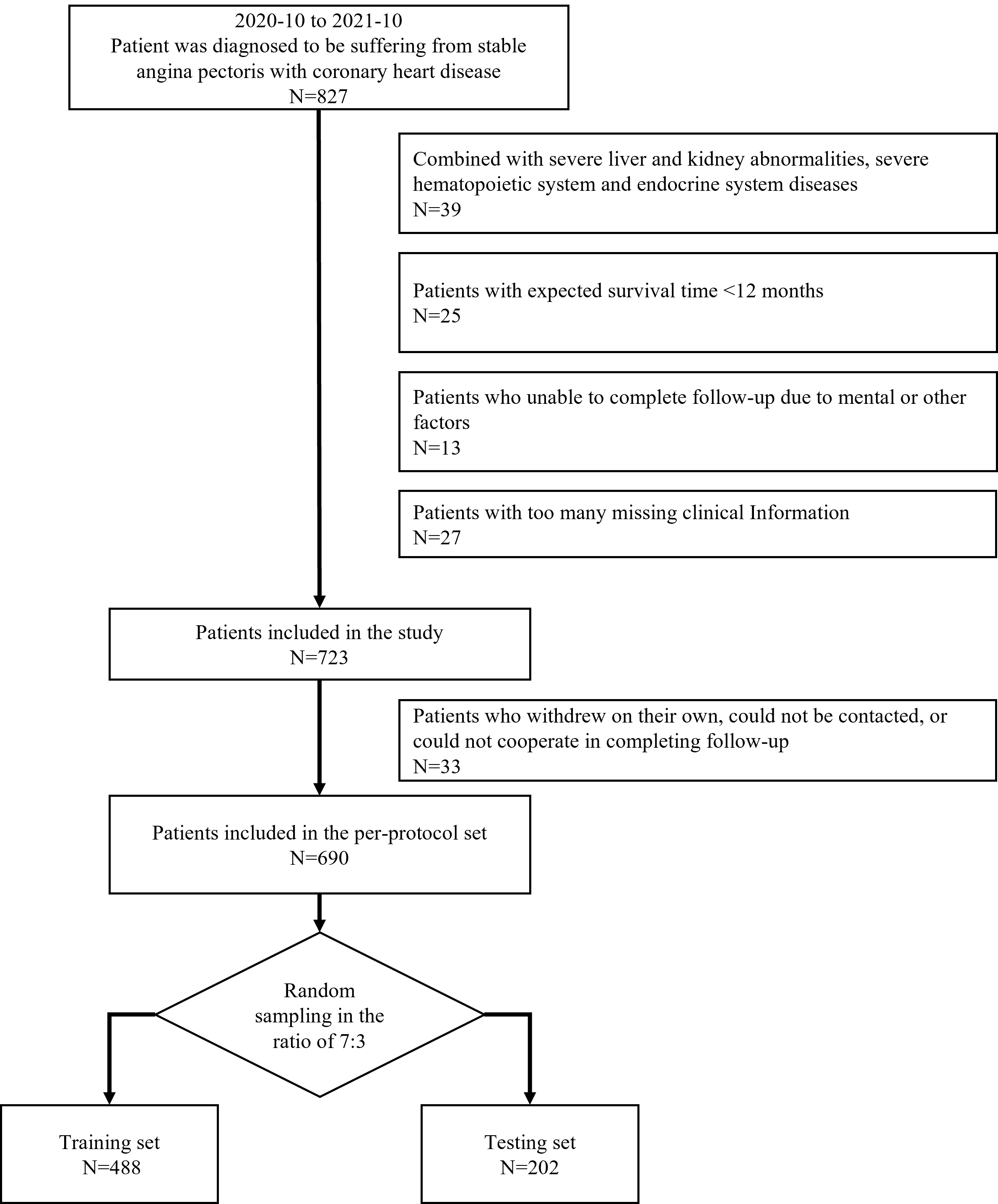

Supplement: Supplementary file 5 [file Image1.TIF]
